# Healthy body, healthy mind: Long-term mutual benefits between classroom and sport engagement in children from ages 6 to 12 years

**DOI:** 10.1016/j.pmedr.2021.101581

**Published:** 2021-09-30

**Authors:** Marie-Josée Harbec, Gary Goldfield, Linda S. Pagani

**Affiliations:** a School of Psycho-Education, University of Montreal, Canada; b School Environment Research Group, University of Montreal, Canada; c Healthy Active Living and Obesity Research Group, Children's Hospital of Eastern Ontario, Ottawa, Canada; d Sainte-Justine’s Pediatric Hospital Research Center, University of Montreal, Canada

**Keywords:** Sport participation, Academic performance, Classroom engagement, Longitudinal study, Child development

## Abstract

Past research suggests that the relationship between health and schooling is axiomatic. Physical activity, including sport participation, putatively facilitates school performance. However, the direction of this link lacks clarity. We examine the mutual links between sport and classroom engagement in 452 boys and 514 girls from ages 6 to 12 years. Participants are from the Quebec Longitudinal Study of Child Development, a prospective-longitudinal birth cohort. First, trajectories of classroom engagement from ages 6 to 10 years, assessed by teachers, were generated using latent class analysis. Second, analyses of covariance (ANCOVAs) compared leisure time physical activity, self-reported by children at age 12 years, across trajectories of classroom engagement. Third, ANCOVAs compared classroom engagement, measured by teachers at age 12 years, across trajectories of extracurricular sport between ages 6 to 10 years. We identified two classroom engagement trajectories: ‘High’ (77%) and ‘Moderate’ (23%). For girls, being in the ‘High’ trajectory predicted significantly higher levels of physical activity (F(1, 966) = 5.21, p < .05). For boys, being in the ‘Consistent participation’ extracurricular sport trajectory predicted significantly higher levels of classroom engagement (F(1, 966) = 6.29, p < .05). Our analyses controlled for pre-existing individual and family factors. Our findings suggest that sport participation and engaged classroom behavior positively influence each other during childhood. They support the pertinence of investing financial resources in youth intervention so that children can develop their potential both in sporting contexts and in the classroom to foster optimal growth and development.

The relationship between health and achievement is very consistent (Zajacova and Lawrence, 2018). Kindergarten children must develop important skills that will enable them to successfully meet the challenges of formal schooling. To optimize their potential children must, among other things, work cooperatively, independently, and with self-discipline. Desirable engagement behaviors promise a developmental course toward later academic, personal, and workplace success (Archambault et al., 2013, Cunha et al., 2006, Fitzpatrick, 2012, Pagani et al., 2012, Shogren et al., 2017).

From a population-health perspective, physical activity through sport and school participation are two important components of health promotion in youth (Griebler et al., 2017, Pascoe et al., 2020). These also represent valuable targets for intervention because they are concrete, malleable, and seek the development of effortful participation skills (Lubans et al., 2016). Thus, generating better understanding of the relationship between sporting and school contexts would be beneficial.

In children, physical activity is most often practiced in an extracurricular sport context (Eime et al., 2013). It could be competitive or not, structured by a coach/instructor and played as a team or individually. One study has shown that 77% of Canadian children aged 5 to 19 years engage in organized physical activity and sport (Canadian Fitness and Lifestyle Research Institute [CFLRI], 2019). However, engagement peaks in the beginning of high school before remaining stable or declining five years later (Kemp et al., 2019, Zimmerman-Sloutskis et al., 2010). Although parents enlist boys and girls equally in childhood extracurricular sport (CFLRI, 2019), the percentage of boys who meet the recommended 60 min of moderate-to-vigorous physical activity daily is almost twice that of girls (LeBlanc et al., 2015).

Behavior at school is one important factor underlying academic performance (Fitzpatrick and Pagani, 2013, Fredricks, 2015). Classroom engagement, more specifically, represents an ecological indicator of mental governance (i.e. executive functions) and reflects ability to follow rules and mobilize task-oriented behavior in the classroom. Compared to boys, girls tend to show higher active classroom participation/involvement (Pagani et al., 2012). Remarkably, this gender distribution is the opposite of participation in physical activity.

The literature suggests an association between sport and classroom engagement in youth. Systematic reviews conclude that physical activity, including sport, is beneficial for classroom behavior, academic achievement, and cognitive functioning, such as levels of attention and concentration (Álvarez-Bueno et al., 2017, Bidzan-Bluma and Lipowska, 2018, de Greeff et al., 2018, Donnelly et al., 2016). Regular participation in physical activity may improve child behavior in the classroom over the long term. In fact, regular participation in leisure-time kindergarten physical activity with a coach or instructor forecasts achievement at sixth and fourth grade (Gonzalez-Sicilia et al., 2019, Piché et al., 2015). Early participation in physical activity, including sport, may facilitate the development of socioemotional, motivational, and effortful child behavior (Lubans et al., 2016). These are fundamental of classroom engagement.

Sport participation represents an activity of daily living in the context of the Positive Youth Development (PYD) framework (Lerner et al., 2018). By experiencing mutually beneficial relations with significant others and social institutions, youth are more likely to project themselves on a path of positive contributions toward themselves, their families, and their communities. The PYD framework suggests that sporting contexts and classrooms represent protective environments characterized by caring relationships with adults (teachers/coaches/instructors) and positive interactions with peers. These foster personal, social, and physical benefits for youth (Holt et al., 2017, Holt et al., 2020, Lerner et al., 2018). In the same vein, according to flourishing theory, an optimal life represents a state of positive emotions, engagement, relationships, and experiencing motivation and accomplishment (Seligman, 2011). Much like PYD, flourishment is cultivated by valuing one’s strengths and talents as resources.

Akin to classroom settings, sporting activities can encourage the development of physical, behavioral, or cognitive skills needed to manage challenges in everyday life (Holt et al., 2017, Holt et al., 2020). Interactions between children and peers in both contexts can enable often neglected skills, such as self-discipline, self-control in conflict/goal obstruction, self-awareness, and teamwork (Felfe et al., 2016). Peers and adults are meant to be role models for honing skills (Piché et al., 2012, Piché et al., 2015). Youth engagement in the two settings could also improve confidence in the ability to perform specific tasks (Lubans et al., 2016). Preliminary longitudinal research suggests that multiple skills and benefits are transmitted between both childhood contexts, thus reinforcing commitment values (Piché et al., 2012, Piché et al., 2015) and positive youth development which leads to flourishment (Lerner et al., 2018, Seligman, 2011).

Past research exploring the relationship between sport participation and classroom engagement is not without limitations. First, few studies used a longitudinal design, with repeated measures of these two characteristics throughout childhood. Second, childhood sport and school engagement has been significantly under-studied. Since there is a developmental continuity in lifestyle habits during childhood (Archambault et al., 2013, Howie et al., 2020, Rovio et al., 2018), more information on early habits might optimize their persistence into adulthood. Finally, and most importantly, previous studies have mostly examined one-way associations of sport on academic characteristics. Few studies have examined whether these two variables influence each other over time.

The current study will attempt to overcome the limitations of previous research mentioned above. Therefore, using multiple measures and longitudinal data sources, the purpose of this paper is to prospectively examine the mutual relationship between extracurricular sport and classroom engagement from ages 6 to 10 years on similar lifestyle factors at age 12 years. Unlike most previous studies, we will conduct a prospective and developmental approach, using more than one data point to facilitate internal and external validity. As a second innovation, we will treat boys and girls as distinct populations, given that they experience risk and protective factors differently (Howie et al., 2020). To best ensure unbiased relationships, we will also control for multiple confounders in early childhood. We expect that childhood sport engagement will distinctly predict classroom engagement at age 12 years and that childhood classroom engagement will predict engagement in self-reported leisure time physical activity at age 12 years, thus reflecting reciprocal longitudinal associations of mutual benefits in boys and girls. Indeed, much like classroom teachers, coaches and instructors typically encourage task-oriented behavior (i.e. focus or play the sport or game). This is where sport and schooling contexts converge (Fitzpatrick and Pagani, 2013).

## 1 Methods

### 1.1 Participants

This IRB approved study met the institution’s guidelines for protection of human subjects concerning their safety and privacy. Participants are from the Québec Longitudinal Study of Child Development (QLSCD) birth cohort. The QLSCD originates from a randomly selected, stratified sample of 2 837 infants Recorded on the provincial birth register, the target population for the QLSCD includes all singletons born between spring 1997/spring 1998 in Quebec (Canada), with French/English-speaking mothers living in Quebec, excluding mothers living in native territories. An explanatory brochure was sent to targeted families. Parents were then telephoned to schedule the first in-person interview with interested and eligible families. At the inception of the longitudinal component, in 1998, 93 children were deemed ineligible and 172 were untraceable owing to incorrect coordinates. Of the 2 572 remaining children, 349 parents were unreachable or refused participation. At age five months, 2223 infants (and their families) with parental consent were selected for longitudinal follow-up, representing 82% of the eligible target population. Of these, 39% were firstborn. A chief epidemiological strength of the QLSCD is that it comprises a large representative sample of typically developing children.

In this study, we used a subsample of 966 children who had complete data on classroom engagement at age 6 years (see Table 1). Classroom engagement and sport participation were measured annually from ages 6 to 12 years (2004–2010).

**Table 1:** Socio-demographic characteristics of the included sample, n = 966.

Variables	M (SD)	%
Age		
	6.1 years (0.3)	
Sex		
Male Female		46.8 53.2
Language spoken at home		
Only French Only English Other		87.2 6.9 5.9
Family income		
Sufficient Insufficient Very insufficient		85.9 9.0 5.1
Family configuration		
Intact Stepfamily Single-parent		72.5 13.0 14.5
Type of school		
Public Private Other		96.1 3.8 0.1

Notes. M = mean; SD = standard deviation.

### 1.2 Measures: predictor variables (at ages 6, 7, 8, and 10 years)

*Classroom engagement trajectories.* Teachers reported upon 11 items that capture behaviors indicative of adaptive and cognitive control: child plays and works cooperatively; follows rules; demonstrates self-control; shows self-confidence; listens attentively; follows directions; completes work on time; works independently; takes care of school materials; works neatly and carefully; can solve daily problems on his own. Scores were derived from the mean of each item responses (1 to 3), with higher values indicating a higher degree of classroom engagement. Alphas ranged from 0.89 to 0.91. The validity and reliability of this classroom engagement scale has been shown in prior publications on child development and academic adjustment (Fitzpatrick and Pagani, 2013, Pagani et al., 2010a, Pagani et al., 2010b).

*Extracurricular sport engagement trajectories.* In this study, we used previously validated childhood trajectories of participation in extracurricular sport generated in Brière et al. (2020) using longitudinal latent class analysis. The ‘Consistent Engagement’ trajectory included children with elevated probability of participation from ages 6 to 10 years (see Table 2 for %). The ‘Low-Inconsistent Engagement’ trajectory included children who did not participate or participated only once or twice, generally in late childhood.

**Table 2:** Descriptive statistics for study variables.

	Boys			Girls		
	M (SD)	Categorical variables (%)	Range	M (SD)	Categorical variables (%)	Range
*Predictors (ages 6 to 10 years)*						
Classroom engagement 1 = moderate 2 = high	– – –	– 31.6 68.4	1 – 2 – –	– – –	– 16.0 84.0	1 – 2 – –
Extracurricular sport 1 = low-inconsistent participation 2 = consistent participation	– – –	– 37.0 63.0	1 – 2 – –	– – –	– 32.5 67.5	1 – 2 – –
*Outcomes (age 12 years)*						
Classroom engagement	2.72 (0.3)	–	1.85 – 3	2.80 (0.2)	–	1.85 – 3
Leisure time physical activity 1 = no 2 = yes	– – –	– 12.2 87.8	1 – 2 – –	– – –	– 15.0 85.0	1 – 2 – –
*Control variables*						
Child BMI (fractional rank %; age 2)	52.5 (27.7)	–	0 – 100	47.8 (28.6)	–	0 – 100
Sport participation (age 5) 0 = never 1 = any participation	– – –	– 50.0 50.0	0 – 1 – –	– – –	– 37.9 62.1	0 – 1 – –
Mathematical skills (NKT; age 6)	13.2 (3.3)	–	3 – 18	13.3 (3.3)	–	4 – 18
Verbal competence (PPVT; age 6)	80.5 (17.1)	–	19 – 121	80.2 (17.3)	–	0 – 130
Maternal education (5 mo) 0 = finished high school 1 = did not finish high school	– – –	– 83.9 16.1	0 – 1 – –	– – –	– 82.5 17.5	0 – 1 – –
Family functioning (age 1.5) 0 = below or in the median 1 = above the median	– – –	– 57.5 42.5	0 – 1 – –	– – –	– 60.7 39.3	0 – 1 – –
Family configuration (age 2) 0 = two-parent 1 = single-parent	– – –	– 88.9 11.1	0 – 1 – –	– – –	– 89.7 10.3	0 – 1 – –
Family income (age 5) 0 = sufficient 1 = insufficient	– – –	– 83.9 16.1	0 – 1 – –	– – –	– 84.6 15.4	0 – 1 – –

Notes. M = mean; SD = standard deviation; BMI = body mass index; NKT = Number Knowledge Test; PPVT = Peabody Picture Vocabulary Test. Analyses corrected for attrition bias.

### 1.3 Measures: outcome variables (at age 12 years)

*Classroom engagement.* We used the same teacher-reported scale as our predictor variable (*Classroom engagement trajectories*).

*Participation in leisure time physical activity, including sport.* Children self-reported on the following item: “In your leisure time at school, at home or elsewhere, do you do one (or more) physical activities? (These activities can be structured or unstructured. For example, you can play sport, do outdoor activities, exercise, dance or just go for a walk.)” Response options included 1 = no and 2 = yes.

### 1.4 Measures: individual and family control variables (from age 5 months to 6 years)

Individual characteristics include body mass index (BMI) at age 2 years using percentile ranks, sport participation at age 5 years (0 = never and 1 = any participation), mathematical skills at age 6 years (measured individually by trained professionals using the Number Knowledge Test [abridged version]; Okamoto and Case, 1996), and verbal competence at age 6 years (measured individually by trained professionals using the Peabody Picture Vocabulary Test [French adaptation]; Dunn et al., 1993). The French version has been standardized and is highly correlated with other French vocabulary and intelligence tests. Family characteristics include maternal education at age 5 months (0 = finished high school and 1 = not), parent-reported family functioning at age 1.5 years (with lower scores revealing that a family is functional), family configuration at age 2 years (0 = two-parent and 1 = single-parent), and family income at age 5 years (0 = sufficient and 1 = insufficient, as defined by the Canadian low-income cut-off of that year provided by Statistics Canada).

### 1.5 Data analytic procedure

Our aim is to prospectively examine whether extracurricular sport predicts classroom engagement and whether classroom engagement predicts extracurricular sport at age 12 years in boys and girls. Developmental trajectories of classroom engagement at ages 6, 7, 8, and 10 years were generated by using longitudinal latent class analysis (Mplus v.7.1) which offers the possibility to examine whether there are several subgroups in a given population and if there are differences between these subgroups with respect to a given individual characteristic (Nagin, 2005). Trajectories of sport and classroom engagement served as predictors of similar outcomes, using analyses of covariance (ANCOVAs). First, ANCOVAs stratified by sex compared leisure time physical activity at age 12 years by different trajectories of classroom engagement (SPSS v.25). Second, ANCOVAs stratified by sex compared classroom engagement at age 12 years by two different trajectories of extracurricular sport (SPSS v.25). The level statistical significance was set at 0.05 (5%) for this study.

As with any longitudinal study, incomplete data required an attrition analysis to compare the participants with varying incomplete data to participants with complete data from our sample (see Supplemental file). We used SPSS v.25 for multiple imputation to correct for response and attrition bias (Cummings, 2013; see Supplemental file for more details).

## 2 Results

### 2.1 Descriptive statistics

Table 2 reports descriptive statistics stratified by sex for all study variables. From ages 6 to 10 years, 63% of boys had a consistent participation in extracurricular sport and 68% of them showed a high classroom engagement. At age 12 years, 88% of boys participated in leisure time physical activity (including sport) and they had a mean score of 2.7 (on 3) for classroom engagement. As for girls, from ages 6 to 10 years, 68% of them had a consistent participation in extracurricular sport and 84% of them showed a high classroom engagement. At age 12 years, 85% of girls participated in leisure time physical activity and they had a mean score of 2.8 for classroom engagement.

Bivariate correlations stratified by sex among study variables are documented in Table 3. For both boys and girls, extracurricular sport from ages 6 to 10 years is positively correlated with classroom engagement at age 12 years (Pearson *r* = 0.22 and 0.17, respectively, p < .01). Classroom engagement from ages 6 to 10 years is positively correlated with leisure time physical activity (including sport) at age 12 years for girls (Pearson *r* = 0.11, p < .05), but not for boys.

**Table 3:** Correlations among study variables.

	Variables	2	3	4	5	6	7	8	9	10	11	12
*Boys*	1. Extracurricular sport (ages 6 to 10)	0.24**	0.22**	0.14*	0.03	0.40**	0.21**	0.22**	−0.32**	−0.10*	−0.14**	−0.22**
	2. Classroom engagement (ages 6 to 10)		0.41**	0.09	0.03	0.14**	0.30**	0.27**	−0.19**	−0.06	−0.08	−0.17**
	3. Classroom engagement (age 12)			0.18**	0.02	0.20**	0.15**	0.21**	−0.23**	−0.04	−0.07	−0.16*
	4. Leisure time PA (age 12)				−0.01	0.05	0.04	0.07	−0.08	−0.02	−0.06	−0.01
	5. Child BMI (age 2)					0.08	0.03	0.05	−0.07	0.003	0.06	0.04
	6. Sport participation (age 5)						0.19**	0.18**	−0.24**	−0.06	−0.12*	−0.20**
	7. Mathematical skills (age 6)							0.36**	−0.22**	−0.03	0.01	−0.11*
	8. Verbal competence (age 6)								−0.12*	−0.12*	−0.03	−0.22**
	9. Maternal education (5 months)									0.09	0.18**	0.21**
	10. Family functioning (age 1.5)										0.12*	0.05
	11. Family configuration (age 2)											0.30**
	12. Family income (age 5)											1

	Variables	2	3	4	5	6	7	8	9	10	11	12

***Girls***	1. Extracurricular sport (ages 6 to 10)	0.19**	0.17**	0.16*	0.03	0.48**	0.19**	0.17**	−0.24**	−0.13**	−0.10*	−0.25**
	2. Classroom engagement (ages 6 to 10)		0.35**	0.11*	−0.04	0.12**	0.37**	0.29**	−0.17**	−0.07	−0.15**	−0.18**
	3. Classroom engagement (age 12)			0.18**	0.06	0.13*	0.24**	0.21**	−0.21**	−0.07	−0.05	−0.15**
	4. Leisure time PA (age 12)				0.02	0.07	0.15**	0.12*	−0.01	−0.07	−0.004	−0.05
	5. Child BMI (age 2)					−0.02	0.01	0.03	0.05	−0.02	0.01	0.03
	6. Sport participation (age 5)						0.17**	0.17**	−0.24**	−0.06	−0.09	−0.27**
	7. Mathematical skills (age 6)							0.50**	−0.17**	−0.12*	−0.09*	−0.19**
	8. Verbal competence (age 6)								−0.17**	−0.16**	−0.08	−0.12*
	9. Maternal education (5 months)									0.07	0.12**	0.27**
	10. Family functioning (age 1.5)										0.04	0.09
	11. Family configuration (age 2)											0.30**
	12. Family income (age 5)											1

Notes. PA = physical activity; BMI = body mass index. Analyses corrected for attrition bias. *p < .05, **p < .01.

### 2.2 Classroom engagement trajectories

**Fig. 1:**
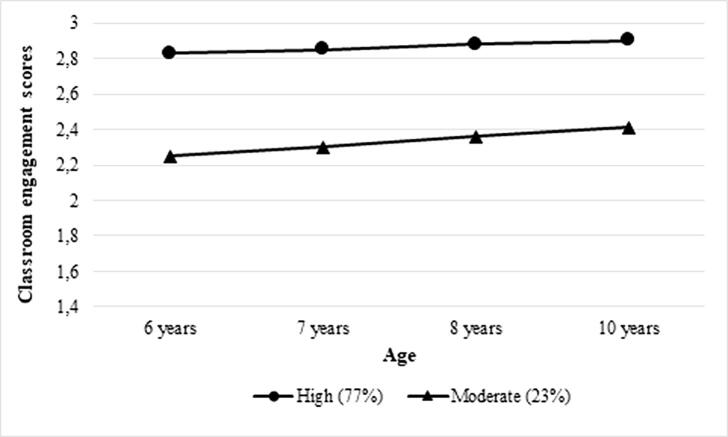
Trajectories of classroom engagement.

We estimated models with 1 to 4 classes without predictors and outcomes. Multiple criteria were used to select the optimal trajectory solution including information criteria; likelihood ratio tests testing the improvement of solutions with k classes vs. k-1 classes; and substantive reasoning (see Table 4; Nagin, 2005). Based on these criteria, we thus selected the 2-class model to represent our classroom engagement developmental trajectories. The two identified developmental trajectories are illustrated in Fig. 1. Seventy-seven percent of the sample showed high levels of classroom engagement from ages 6 to 10 years. We labeled this trajectory ‘High’. Twenty-three percent of children showed lower levels of classroom engagement from ages 6 to 10 years. We labeled this trajectory ‘Moderate’.

**Table 4:** Model fit statistics for 1–4 latent classes.

	Classes			
	1	2	3	4
AIC	1684.788	290.060	130.374	1.402
BIC	1714.027	333.919	188.852	74.499
Adjusted BIC	1694.971	305.335	150.740	26.860
VLMR (p)	–	0.0000	0.1341	0.0553
LRT (p)	–	0.0000	0.1453	0.0604
Entropy	–	0.907	0.828	0.852
Number and proportions of each class (C)	C1: n = 966 (100 %)	C1: n = 225 (23.3 %) C2: n = 741 (76.7 %)	C1: n = 660 (68.3 %) C2: n = 177 (18.3 %) C3: n = 129 (13.4 %)	C1: n = 115 (11.9 %) C2: n = 103 (10.7 %) C3: n = 676 (69.9 %) C4: n = 72 (7.5 %)

Notes. AIC = Akaike information criteria; BIC = Bayesian information criteria; VLMR = Vuong-Lo-Mendell-Rubin likelihood ratio test; LRT = Lo-Mendell-Rubin adjusted LRT test.

### 2.3 Leisure time physical activity (including sport) at age 12 years as an outcome of classroom engagement trajectories

We then examined associations stratified by sex between teacher-reported classroom engagement trajectories and subsequent leisure time physical activity, as reported by children themselves at age 12 years. Our analyses controlled for child BMI (age 2 years), early child sport participation (age 5 years), maternal education, family functioning, configuration, and income.

Exclusively for girls, results revealed adjusted omnibus differences on the outcome assessed. For girls, being in the ‘High’ classroom engagement trajectory predicted significantly higher levels of leisure time physical activity compared to girls in the ‘Moderate’ classroom engagement trajectory (F(1, 966) = 5.21, p < .05).

### 2.4 Classroom engagement at age 12 years as an outcome of extracurricular sport trajectories

Finally, we proceeded to examine associations stratified by sex between parent-reported extracurricular sport trajectories and subsequent classroom engagement, as reported by teachers at age 12 years. Our analyses controlled for mathematical skills and verbal competence at age 6 years, maternal education, and family functioning, configuration, and income.

Exclusively for boys, results revealed adjusted omnibus differences on the outcome assessed. For boys, being in the ‘Consistent participation’ extracurricular sport trajectory predicted significantly higher levels of classroom engagement compared to boys in the ‘Low-inconsistent’ physical activity trajectory (F(1, 966) = 6.29, p < .05).

## 3 Discussion

As a test of the old adage, *Mens sana in corpore sano*, this study sought to examine the prospective long-term mutual links between engagement in sporting and classroom activities in middle childhood. Past research suggests mutual benefits through reciprocal relations (Álvarez-Bueno et al., 2017, Bidzan-Bluma and Lipowska, 2018, de Greeff et al., 2018, Donnelly et al., 2016, Piché et al., 2012, Piché et al., 2015). We found distinct long-term, mutual, and positive relationships between child classroom and sport engagement for boys and girls. To our knowledge, this is the first study to use a prospective and developmental approach to examine the reciprocal relationship between classroom and sport engagement in school-aged children.

First, we found that, compared to girls who were reported as being less engaged by their teachers, girls who completed work on time and independently, demonstrated self-confidence, and were able to solve daily problems on their own were more likely to take part in physical activity in their leisure time, including sport. Practicing frequent physical activity, even during leisure time, requires self-discipline, especially during adolescence and adulthood (Bogg and Roberts, 2004, Rhodes and Boudreau, 2017, Wilson and Dishman, 2015). Thus, girls who have developed and demonstrated self-discipline in the classroom may find it easier to engage in sport at age 12 years, a developmental period where such interest declines (CFLRI, 2019, Kemp et al., 2019, Zimmerman-Sloutskis et al., 2010). It could also be that girls with higher classroom engagement skills have an easier time interacting with peers during sporting activities, thus promoting more frequent participation (Fitzpatrick and Pagani, 2013).

We found no significant associations between childhood classroom engagement and self-reported participation in leisure time physical activity, including sport, for boys at age 12 years. We could speculate that regardless of their behavior in class, boys are likely to be more physically active than girls, creating an explanatory ceiling effect for boys (Kohl and Hobbs, 1998, LeBlanc et al., 2015, Statistics Canada, 2016, Zimmerman-Sloutskis et al., 2010).

Second, we examined developmental trajectories of extracurricular sport in relation to classroom engagement. We found that, compared to boys who rarely took part in extracurricular sport, boys who showed more consistent engagement in sport were better at playing and working cooperatively, following rules and directions, and demonstrating self-control and self-confidence in the classroom. One plausible mechanism to explain this result would be that boys learn a lot and develop various skills in structured physical activity contexts, skills that can then be transferred in a classroom setting (Felfe et al., 2016, Holt et al., 2017, Lubans et al., 2016, Piché et al., 2015). Listening to your coach, playing cooperatively with your peers, having a good team spirit, being disciplined, and controlling your emotions are all skills that are put into practice in a sporting context. These skills are very similar to those valued by teachers and peers in a classroom setting. Coaches and instructors might have helped boys to stay on task (i.e. focus or play the sport or game) and this likely relates to what they are supposed to be doing in class (Fitzpatrick and Pagani, 2013). Moreover, boys could have gained social experience through the sporting environment, frequently based on positive/supportive relationships with/between adults, peers, and parents (Holt et al., 2017, Holt et al., 2020).

For girls, we found no significant associations between childhood extracurricular sport and teacher-reported classroom engagement at age 12 years. One possible explanation could be that girls naturally have more facility paying attention and showing active involvement/participation in class (Gurian and Stevens, 2004, Pagani et al., 2012), thus creating a ceiling effect. Boys, on the other hand, respond to more active learning opportunities in elementary school than do girls (Carrier, 2009).

This study is not without limitations. First, even though our statistical models were adjusted with pre-existing and concurrent controls, we cannot imply causal interpretations. Nonetheless, the use of a longitudinal design allowed us to determine the temporal sequence of the variables of interest. Second, measures of participation in extracurricular sport and leisure time physical activity were derived from less detailed parent- and self-reported epidemiological population health data, especially for the outcome at age 12 years. Complementary instruments that deepen our understanding of sport and classroom engagement associations would have been ideal. Finally, as with any longitudinal study, we experienced attrition in our sample. To minimize potential bias from missing data on our findings, we performed multiple imputation, considered an exemplary method for treating missing data given that it provides a realistic estimate of standard error terms (Cummings, 2013). Despite these limitations, we consider several strengths in our study, such as the large representative sample and prospective design, use of trajectories over point prediction, validated measures, and innovative treatment of childhood lifestyle characteristics.

In conclusion, there are important implications for policy, practice, research, and public health promotion strategies. Childhood sport participation and classroom engagement positively influence one other. By valuing children’s strengths and talents as resources, we encourage their state of positive emotions and relationships, and their sense of accomplishment (Lerner et al., 2018, Seligman, 2011). Physical and cognitive activity through sport represent two key components of health promotion in youth (Griebler et al., 2017, Pascoe et al., 2020). Both predict population health, which is strongly related to economic growth of any nation (Cunha et al., 2006).

Intervention programs that target young children are amongst the most cost-effective (Elango et al., 2016). Our findings suggest benefits of investing financial resources in youth intervention so that children can develop their full potential both in the classroom and in extracurricular contexts. As a society, our findings support the development of programs that offer to our youth contexts/opportunities that foster positive and optimal developmental change. An interesting avenue for practice that offers positive influences on youth is to offer child development training to sport personnel so that they can intervene directly with youth under their charge. Project SCORE (https://www.projectscore.ca/en/) and *Pour 3 Points* (https://pour3points.ca/en/) represent two examples of programs that train sport coaches to help youth develop skills to succeed in school and in life in a general. Finally, our findings suggest that sporting contexts can potentially facilitate youth flourishment.

## 4 Disclosure of funding and conflicts of interest

This work was supported by the Social Sciences and Humanities Research Council and by Sport Canada (MJH, scholarship number 752-2019-1325; LSP and GG, grant number 435‐2017‐0784). In addition to acknowledging the funding to these specific secondary analyses, we acknowledge the generous funding provided by the Fondation Lucie et André Chagnon, the Institut de la Statistique du Québec, the Ministère de l'Éducation et de l'Enseignement supérieur, the Ministère de la Famille, the *Ministère du Travail, de l’Emploi et de la Solidarité*, the Institut de recherche Robert-Sauvé en santé et en sécurité du travail, the Centre hospitalier universitaire Sainte-Justine, and the Ministère de la Santé et des Services sociaux du Québec. These original sponsors funded the larger public data set that constitutes the original Quebec Longitudinal Study of Child Development. Source: Data compiled from the final master file ‘E1-E20′ from the Quebec Longitudinal Study of Child Development (1998–2017), ©Gouvernement du Québec, Institut de la statistique du Québec. The authors have no conflicts of interest to declare.

## CRediT authorship contribution statement

**Marie-Josée Harbec:** Conceptualization, Methodology, Formal analysis, Writing – original draft, Writing – review & editing, Visualization. **Gary Goldfield:** Writing – review & editing, Funding acquisition. **Linda S. Pagani:** Conceptualization, Methodology, Writing – original draft, Supervision, Funding acquisition.

## Declaration of Competing Interest

The authors declare that they have no known competing financial interests or personal relationships that could have appeared to influence the work reported in this paper.
