# Supplementary material for: Healthy body, healthy mind: Long-term mutual benefits between classroom and sport engagement in children from ages 6 to 12 years
Source: Prev Med Rep. 2021 Sep 30;24:101581. doi: 10.1016/j.pmedr.2021.101581 (PMC8683901; doi:10.1016/j.pmedr.2021.101581)
Supplement: Supplementary data 1 [file mmc1.docx]

**SUPPLEMENTAL FILE**

**Attrition analysis**

This study required follow-up data from several sources and waves. These correspond to the predictors, outcomes, and potential confounders in early childhood. As with any longitudinal study, incomplete data required an attrition analysis to compare the participants with varying incomplete data on control variables to participants with complete data on control variables from our sample. Using independent sample t-tests, one significant bivariate difference was found. Compared with the nonretained cases, our retained sample at age 12 years had higher verbal competence skills at age 6 years ($\overset{-}{x}$ = 81.5 vs 78.5; *t*_931_ = 2.42; p *<* .05). There were no significant between-group differences in the other potential confounders in early childhood.

**Multiple imputation**

We used SPSS v.25 for multiple imputation to correct for response and attrition bias (Cummings, 2013). Using a stochastic algorithm, missing observations are imputed based on available complete data on auxiliary variables, creating multiple datasets that are copies of the original complete data. The algorithm generates slightly different values for each imputed measure across the multiple datasets. The additional variance caused by differences in imputed values between the various copies reflects the uncertainty of the imputation and is added as a correction to the analyses (Cummings, 2013). Our analyses were conducted with 20 imputed data sets.

**Bivariate differences between boys and girls**

Using independent sample t-tests, some significant bivariate differences were found between boys and girls among the potential confounding variables in early childhood. Compared to boys, girls had a lower percentile rank in BMI at age 2 years ($\overset{-}{x}$ = 47.8 vs 52.5; *t*_966_ = -2.48; p *<* .05) and had a higher score on sport participation at age 5 years ($\overset{-}{x}$ = 0.62 vs 0.50; *t*_966_ = 3.76; p *<* .01). There were no significant between-group differences in the other potential confounders in early childhood.
